# Comparative Analysis of Bacterial Community Composition and Structure in Clinically Symptomatic and Asymptomatic Central Venous Catheters

**DOI:** 10.1128/mSphere.00146-17

**Published:** 2017-09-27

**Authors:** Franziska A. Stressmann, Elodie Couve-Deacon, Delphine Chainier, Ashwini Chauhan, Aimee Wessel, Sylvaine Durand-Fontanier, Marie-Christine Escande, Irène Kriegel, Bruno Francois, Marie-Cécile Ploy, Christophe Beloin, Jean-Marc Ghigo

**Affiliations:** a Unité de Génétique des Biofilms, Département de Microbiologie, Institut Pasteur, Paris, France; b Université Limoges, INSERM, CHU Limoges, UMR 1092, Limoges, France; c CHU de Limoges, Service de chirurgie digestive générale et endocrinienne, Limoges, France; d Université de Limoges, Équipe de recherche médicale appliquée, Limoges, France; e Institut Curie, Hôpital, Laboratoire de Microbiologie Médicale, Paris, France; f Institut Curie, Hôpital, Service Anesthésie-Réanimation-Douleur, Paris, France; g INSERM, CIC-1435, Limoges, France; Centers for Disease Control and Prevention

**Keywords:** ecology, bacterial community, biofilm, catheter colonization

## Abstract

Totally implanted venous access ports (TIVAPs) are commonly used catheters for the management of acute or chronic pathologies. Although these devices improve health care, repeated use of this type of device for venous access over long periods of time is also associated with risk of colonization and infection by pathogenic bacteria, often originating from skin. However, although the skin microbiota is composed of both pathogenic and nonpathogenic bacteria, the extent and the consequences of TIVAP colonization by nonpathogenic bacteria have rarely been studied. Here, we used culture-dependent and 16S rRNA gene-based culture-independent approaches to identify differences in bacterial colonization of TIVAPs obtained from two French hospitals. To explore the relationships between nonpathogenic organisms colonizing TIVAPs and the potential risk of infection, we analyzed the bacterial community parameters between TIVAPs suspected (symptomatic) or not (asymptomatic) of infection. Although we did not find a particular species assemblage or community marker to distinguish infection risk on an individual sample level, we identified differences in bacterial community composition, diversity, and structure between clinically symptomatic and asymptomatic TIVAPs that could be explored further. This study therefore provides a new view of bacterial communities and colonization patterns in intravascular TIVAPs and suggests that microbial ecology approaches could improve our understanding of device-associated infections and could be a prognostic tool to monitor the evolution of bacterial communities in implants and their potential susceptibility to infections.

**IMPORTANCE** Totally implanted venous access ports (TIVAPs) are commonly used implants for the management of acute or chronic pathologies. Although their use improves the patient’s health care and quality of life, they are associated with a risk of infection and subsequent clinical complications, often leading to implant removal. While all TIVAPs appear to be colonized, only a fraction become infected, and the relationship between nonpathogenic organisms colonizing TIVAPs and the potential risk of infection is unknown. We explored bacteria present on TIVAPs implanted in patients with or without signs of TIVAP infection and identified differences in phylum composition and community structure. Our data suggest that the microbial ecology of intravascular devices could be predictive of TIVAP infection status and that ultimately a microbial ecological signature could be identified as a tool to predict TIVAP infection susceptibility and improve clinical management.

## Abstract (precis)

Totally implanted venous access ports (TIVAPs) are commonly used implants for the management of acute or chronic pathologies. Although their use improves the patient’s health care and quality of life, they are associated with a risk of infection and subsequent clinical complications, often leading to implant removal. While all TIVAPs appear to be colonized, only a fraction become infected, and the relationship between nonpathogenic organisms colonizing TIVAPs and the potential risk of infection is unknown. We explored bacteria present on TIVAPs implanted in patients with or without signs of TIVAP infection and identified differences in phylum composition and community structure. Our data suggest that the microbial ecology of intravascular devices could be predictive of TIVAP infection status and that ultimately a microbial ecological signature could be identified as a tool to predict TIVAP infection susceptibility and improve clinical management.

## INTRODUCTION

The management of acute or chronic pathologies often requires the use of peripheral or central venous catheters, which greatly improve patient health care (1). Nevertheless, since these devices are used as access entry ports to administer nutritive or therapeutic fluids, they are also associated with a risk of bacterial surface contamination, often leading to the formation of bacterial or fungal biofilms that are difficult to eradicate and cause chronic and nosocomial infections (2).

Among central venous catheters used in modern medicine, totally implantable venous access ports (TIVAPs) are commonly used invasive medical devices. TIVAPs are frequently inserted for parenteral nutrition, chemotherapy, and other long-term drug treatments, for periods that can last months or years (1, 3). In addition to contamination that may occur during surgical insertion, repeated use of these long-term implants to inject or draw fluids increases the risk of TIVAP contamination and therefore infections. The high morbidity and substantial risk of complications associated with TIVAP-related infection often lead clinicians to decide on the traumatic and costly removal of devices suspected of infection (4–6).

TIVAP bacterial colonization can originate from bloodstream bacteria in bacteremic patients or from skin bacteria introduced upon repeated punctures with Huber needles to access TIVAP chambers (1, 6). Consistent with external contamination, the most frequently isolated pathogens associated with TIVAP infection correspond to bacteria commonly found on the skin, such as *Staphylococcus aureus* and *Staphylococcus epidermidis* (7–10).

The predominance of skin bacteria suggests that all TIVAPs could be contaminated and colonized by both pathogenic and nonpathogenic microorganisms present on the surface of the skin (11). It has indeed been shown by culture and molecular studies that all implanted devices become colonized at some point (12–14) and that even symptom-free implants without positive microbial culture can harbor bacteria without leading to TIVAP infection (2, 15). While this suggests that TIVAP colonization could be more complex than initially suspected, it also raises the question of whether potential relationships between microorganisms colonizing TIVAP could influence the risk of infection. Unfortunately, microbiology analysis usually targets only TIVAPs suspected of infection and not devices from symptom-free patients considered sterile or free of specific pathogens. Therefore, there is currently no global view of the microbial ecology and the dynamics of bacterial communities developing in TIVAPs. Whether colonization of TIVAPs by nonpathogenic bacteria influences the development of infection remains an unanswered question.

Here, we collaborated with two French hospitals to study the differences between bacterial populations colonizing TIVAP chambers removed from patients receiving long-term cancer therapies. We used both culture–dependent and 16S rRNA-based culture-independent approaches to analyze the bacterial content of TIVAP chambers from patients suspected of infection (here called clinically “symptomatic” TIVAPs) as well as from devices removed from patients without suspicion of infection (here called clinically “asymptomatic” TIVAPs), which are not routinely analyzed in clinical practice. We found a wide range of hospital-associated and environmental bacteria on both symptomatic and asymptomatic chambers, and we compared their compositions and community structures between hospital cohorts. This study presents new findings on the colonization patterns of intravascular devices and on the ecological relationships between the presence of nonpathogenic and pathogenic microorganisms and their ability to colonize catheters.

## RESULTS

### Bicentric sampling of TIVAP catheters from patients suspected or not of catheter-associated infections.

**FIG 1:**
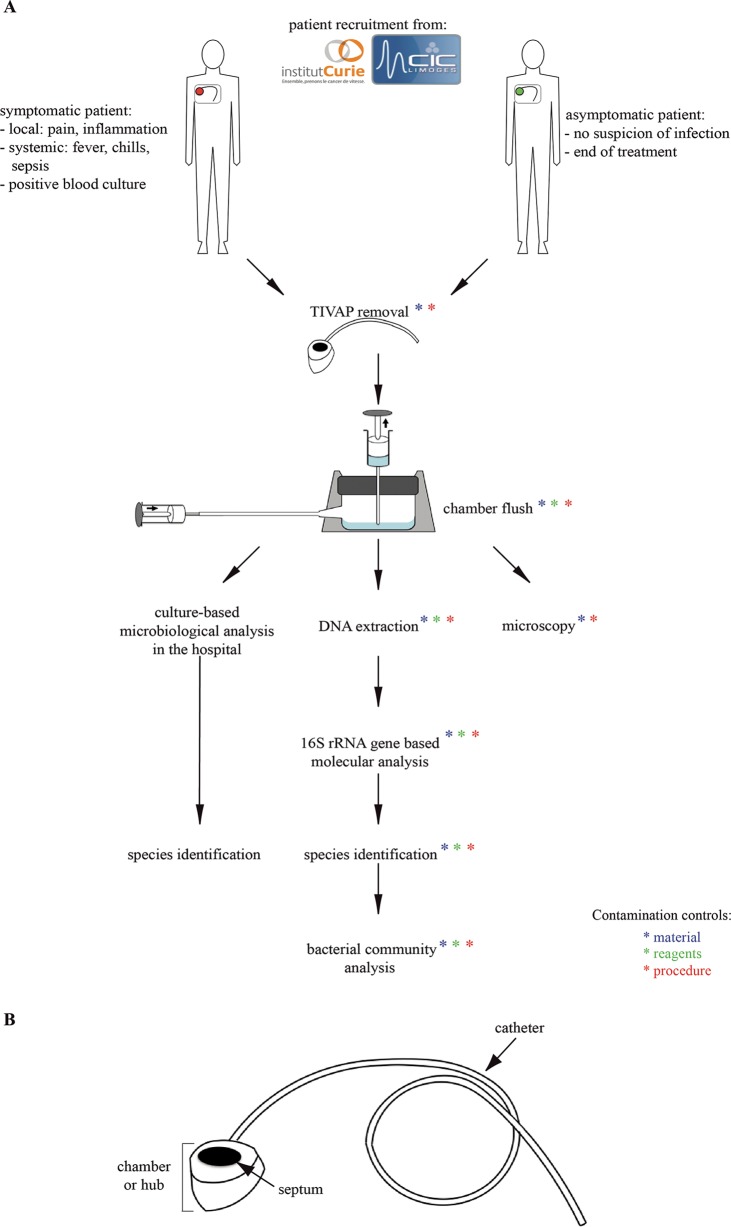
TIVAP catheter collection from patients suspected or not of catheter-related infection from two French hospitals. (A) Study design. Sterile reagents and techniques were used throughout the study, and the protocol was designed to minimize the possibilities for bacterial contamination as much as possible. Furthermore, samples of used reagents (green asterisks), materials (blue asterisks), and blanks (water used as the template in the procedure; red asterisks) as procedure controls were taken at all steps and also submitted to 16S rRNA sequencing for species identification. (B) TIVAP parts. The TIVAP is composed of a subcutaneously implanted chamber/hub (the reservoir) connected to a catheter that is inserted into a central vein (e.g., jugular or subclavian).

Totally implanted venous access ports (TIVAPs) suspected of being contaminated by pathogenic bacteria (here referred to as clinically symptomatic TIVAPs) are systematically removed and subjected to a thorough microbiology analysis. In contrast, TIVAPs removed at the end of treatment without suspicion of infection (clinically asymptomatic TIVAPs) are routinely discarded without analysis. In order to investigate the microbial colonization of TIVAPs implanted in hospitalized patients suspected or not of catheter-associated infection, we developed a collection protocol minimizing bacterial contamination at any point during sample handling (Fig. 1A). Two groups of 10 symptomatic or asymptomatic TIVAPs were collected from two French hospitals (Institut Curie Hospital, Paris, France, and Dupuytren Hospital, Limoges, France, referred to here as Curie and Limoges Hospitals, respectively; total chambers collected, *n* = 40) (see Table S1 in the supplemental material). Due to potential variability and increased risk for postextraction contaminants in TIVAP distal catheter parts, we chose to analyze only the bacterial content of the catheter chamber of each TIVAP (Fig. 1B).

10.1128/mSphere.00146-17.2 TABLE S1  Patient information. Download TABLE S1, PDF file, 0.1 MB. Copyright © 2017 Stressmann et al. 2017 Stressmann et al. This content is distributed under the terms of the Creative Commons Attribution 4.0 International license.

### Culture-independent evidence for environmental bacteria in culture-negative TIVAPs.

To test the potential presence of bacteria in TIVAPs removed from asymptomatic patients, asymptomatic TIVAPs were subjected to routine culture analysis and parallel molecular analysis of bacterial 16S rRNA gene content (clone library generation). Culture analysis detected no bacteria in 9 out of 10 samples from Curie Hospital and in 8 out of 10 samples from Limoges Dupuytren Hospital (Table 1). In contrast, 16S rRNA-based molecular analysis detected bacteria in all asymptomatic TIVAP samples from Curie and Limoges Hospitals (Table 1). Reagents and materials used for molecular processing and blanks (water as the template) for all experimental procedures were also subjected to 16S rRNA sequencing. This led to the identification of 7 species detected in molecular reagents or materials or introduced during DNA extraction or PCR, two of which were also detected in patient samples (Table S2A). When any of these 7 species deemed contaminants from the molecular procedure were identified in patient samples in the study, these species were excluded from all further analysis. Two species were detected in nonimplanted and nonmanipulated TIVAPs but were not detected in any of the patient samples (Table S2B). A total of 14 species were detected as present in the flush solutions used in the hospitals or in handled nonimplanted chambers (Table S2C and D). Four of these species (including *Propionibacterium acnes* and *Ralstonia pickettii*) were also detected in patient samples.

10.1128/mSphere.00146-17.3 TABLE S2  Bacterial species detected as contaminants in reagents or procedures. Species in bold were detected in patient samples; species in lightface were detected in control or blank samples but not in patient samples. (A) Species detected in PCR or DNA extraction reagents or materials or in blank samples (water as the template) used as procedure controls at the Institut Pasteur. When any of these species were detected in a sample, they were excluded from all further analyses. (B) Species detected in nonimplanted and nonmanipulated control TIVAP chambers. (C) Species detected in the solutions used at Curie Hospital to flush the chambers or in manipulated but not implanted chambers. (D) Species detected in the solutions used at Limoges Hospital to flush the chambers or in manipulated but not implanted chambers. Download TABLE S2, PDF file, 0.1 MB. Copyright © 2017 Stressmann et al. 2017 Stressmann et al. This content is distributed under the terms of the Creative Commons Attribution 4.0 International license.

**TABLE 1:** Culture results for infected and noninfected TIVAP chambers from the two hospital cohorts and species (OTU) richness as detected by 16S clone library generation^a^

TIVAP group and sample ID	Culture-isolated organism(s)	No. of CFU/ml	No. of OTUs detected in 16S clone library	No. of clones generated for 16S clone library
Curie symptomatic				
CS-1	Sterile		8	48
CS-2	Staphylococcus aureus*	>1,000	1	48
CS-3	Staphylococcus epidermidis*	200	1	48
CS-4	Staphylococcus epidermidis*	>1,000	1	48
CS-5	Staphylococcus epidermidis*	>1,000	1	48
CS-6	*Candida parapsilosis*	>1,000	5	48
CS-7	Sterile		1	48
CS-8	Staphylococcus aureus*	130	6	45
CS-9	*Staphylococcus xylosus*	<10	4	49
CS-10	*Candida krusei* and Staphylococcus epidermidis*	>1,000	1	48
				
Curie asymptomatic				
CA-1	Sterile		9	48
CA-2	Sterile		10	48
CA-3	Sterile		4	49
CA-4	Sterile		4	49
CA-5	Sterile		6	49
CA-6	Sterile		4	48
CA-7	*Staphylococcus epidermidis*	20	3	48
CA-8	Sterile		7	48
CA-9	Sterile		9	53
CA-10	Sterile		7	48
				
Limoges symptomatic				
LS-1	Staphylococcus epidermidis*, *Bacillus simplex*	1, 1	1	50
LS-2	Sterile		6	47
LS-3	Enterobacter cloacae*	>500	1	50
LS-4	(*Staphylococcus epidermidis* on distal catheter)		2	48
LS-5	Staphylococcus epidermidis*	>500	1	50
LS-6	Escherichia coli*	>500	1	50
LS-7	Staphylococcus aureus*	<50	1	49
LS-8	Staphylococcus aureus*	1	4	48
LS-9	Pseudomonas aeruginosa*	<50	1	49
LS-10	Staphylococcus epidermidis*	<10	2	49
				
Limoges asymptomatic				
LA-1	Sterile		7	52
LA-2	Sterile		4	49
LA-3	Sterile		5	48
LA-4	Sterile		5	50
LA-5	Sterile		3	48
LA-6	*Micrococcus luteus*	1	6	50
LA-7	*Micrococcus luteus*	1	3	50
LA-8	Sterile		8	55
LA-9	Sterile		4	48
LA-10	Sterile		2	50

a Species shown in bold with an asterisk represent the cultured organism that was also the dominant organism detected in the 16S rRNA gene clone library. The bacteria isolated from the TIVAP samples of symptomatic patients were also isolated from the blood cultures.

**FIG 2:**
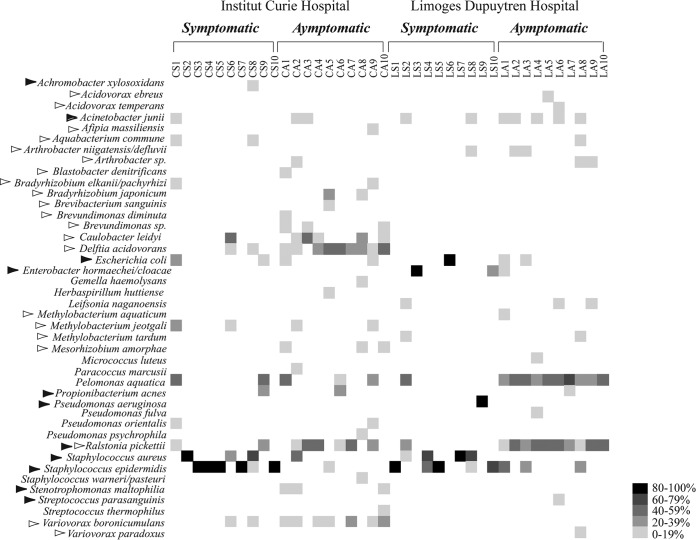
Heat map of species presence and their relative abundance detected in clone libraries from all 40 samples. The gray scale represents the frequency at which each species was detected in the clone library. Black arrowheads designate species generally considered of clinical origin. White arrowheads designate species of environmental origin.

From a total of 42 detected operational taxonomic units (OTUs), 40 different OTUs/bacterial species were detected in asymptomatic TIVAP chambers in both hospitals, 28 in Curie Hospital and 19 in Limoges Dupuytren Hospital (Fig. 2). Although species frequently associated with clinical infections (e.g., *Escherichia coli*, *Enterobacter cloacae*, *S. aureus*, *S. epidermidis*, *Stenotrophomonas maltophilia*, and *Streptococcus parasanguinis*) were detected in some asymptomatic TIVAP samples, the majority of species detected are generally found in the environment. For example, *Aquabacterium* spp., *Bradyrhizobium* spp., *Caulobacter leidyi*, *Delftia acidovorans*, *Methylobacterium* spp., *Mesorhizobium* spp., and *Variovorax paradoxus* (Fig. 2) are commonly isolated from soil and aquatic environments.

These results suggest that the chambers of asymptomatic TIVAPs, although without history of infection, are nevertheless commonly colonized by bacteria that are mostly of environmental origin.

### Environmental bacteria are also detected in culture-positive TIVAPs.

To test which bacteria were present in symptomatic TIVAPs, we carried out culture-based analysis on symptomatic TIVAP chamber flushes, which detected mostly one bacterial species per sample (Table 1). Curie Hospital samples were exclusively colonized by Gram-positive bacteria (*S. aureus*, *S. epidermidis*, and *Staphylococcus xylosus*), with fungal colonization detected in two chambers (Table 1). Samples from Limoges Dupuytren Hospital were colonized by a mixture of Gram-positive (*S. aureus*, *S. epidermidis*, and *Bacillus simplex*) and Gram-negative (*E. coli*, *E. cloacae*, and *Pseudomonas aeruginosa*) bacteria. The bacteria isolated from TIVAP samples of symptomatic patients were also isolated from the blood cultures.

In contrast to culture-based analysis, 16S rRNA analysis enabled us to detect more than 1 species in 40% of Curie and 40% of Limoges Dupuytren Hospital symptomatic samples (Table 1). Moreover, we detected at least one bacterial species in each of the chambers from symptomatic patients that were culture negative (2 from Curie Hospital and 1 from Limoges Hospital).

The dominant organism detected by 16S rRNA PCR and sequencing also corresponded to the bacterial species detected by culture and identified as the infection cause in all chambers but one (CS-9) (Table 1). Besides these bacteria, a total of 20 different bacterial species were detected in symptomatic TIVAP chambers in both hospitals, 15 different species in Curie Hospital and 11 species in Limoges Hospital (Fig. 2). Many of these species can be considered of environmental origin, including *Pelomonas aquatica* (both hospitals), an *Aquabacterium* sp., a *Bradyrhizobium* sp., *Caulobacter leidyi*, *Methylobacterium jeotgali*, and *Variovorax boronicumulans* (Curie Hospital) and *Arthrobacter* spp., *Leifsonia naganoensis*, and *Methylobacterium tardum* (Limoges Dupuytren Hospital). In addition to the species associated with clinical infection detected by culture, 16S rRNA-based analysis identified four additional clinical species, *Achromobacter xylosoxidans* in Curie Hospital, *Acinetobacter junii* in Limoges Hospital, and *Propionibacterium acnes* and *Ralstonia pickettii* in both hospitals (Fig. 2).

Hence, although culture-independent approaches confirmed culture analysis results in the majority of cases, they also indicated the previously unsuspected presence of bacteria from environmental origin in symptomatic TIVAPs.

### Culture-positive TIVAPs reveal higher bacterial load but lower species richness than culture-negative TIVAPs.

Culture-independent 16S rRNA-based analysis detected a higher number of OTUs overall in asymptomatic TIVAPs (40 different species or OTUs) than in symptomatic TIVAPs (20 different species or OTUs). This difference was reflected in mean species richness, which was significantly higher in asymptomatic than in symptomatic samples in Limoges Dupuytren Hospital (asymptomatic, 3.1 ± 2.6 species/sample, range of 2 to 8; symptomatic, 2 ± 1.7 species/sample, range of 1 to 6; *P* = 0.004), as well as in Curie Hospital (asymptomatic, 6.3 ± 2.5 species/sample, range of 3 to 10; symptomatic, 4.7 ± 1.9 species/sample, range of 1 to 8; *P* = 0.008). Moreover, mean species richness in the overall sample set was significantly higher (*P* = 0.0002) in asymptomatic (5.4 ± 2.3 species/sample, range of 2 to 10) than in symptomatic (2.45 ± 2.2 species/sample, range of 1 to 8) samples.

**FIG 3:**
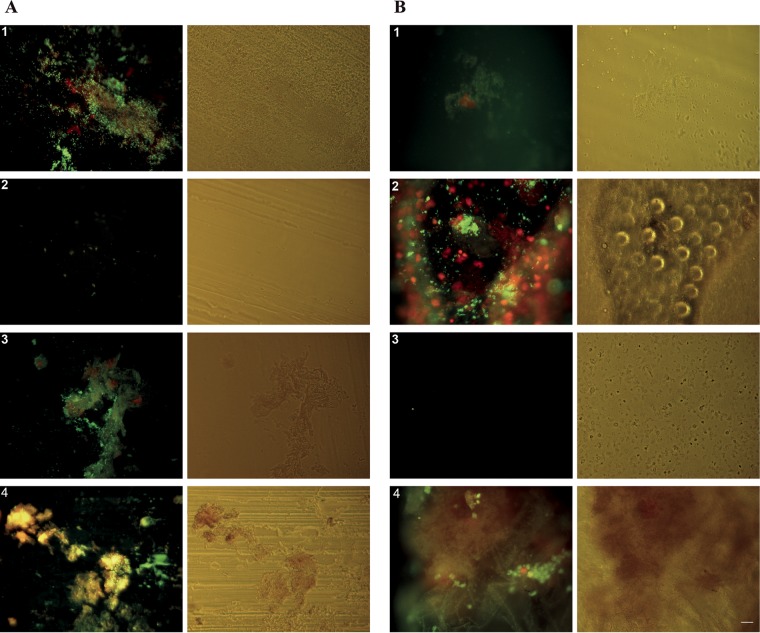
Chamber septum microscopy reveals the presence of bacteria. Cell content on TIVAP septum surfaces from Limoges Hospital (after freeze-thaw treatment) was stained with nucleic acid stains SYTO9 (green, live cells) and propidium iodide (red, membrane-compromised cells). Epifluorescence and corresponding phase-contrast images of chamber septa from four symptomatic patients (A) and of chamber septa from four asymptomatic patients (B). The stain was not exclusive to bacteria; large (>5-μm) staining patterns illustrate the presence of host cell nuclei. Bar, 20 μm.

Based on the concentration of total DNA extracted from asymptomatic TIVAPs for 16S rRNA culture-independent analysis purposes, bacterial content appeared much lower in asymptomatic samples than in symptomatic samples. In a number of asymptomatic samples from Curie (CA-3 and -8) and Limoges (LA-3, -5, -7, and -10) Hospitals, DNA yield was insufficient for performing quantitative real-time PCR; therefore, additional chambers were collected from Limoges Dupuytren Hospital, and the septa were assessed for the presence of bacteria using a pair of nucleic acid stains and epifluorescence microscopy. Elements of size and shape consistent with bacteria could be detected on the septa of all analyzed samples except for one asymptomatic chamber (Fig. 3; Table S3). However, in symptomatic samples, elements consistent with bacterial cells could be seen frequently in 79% (81 of 102) of fields of view analyzed in all samples of this group. In asymptomatic samples, elements consistent with bacterial cells were detected in only 23% (23 of 100) of analyzed fields of view (Table S3). In symptomatic samples, bacterial elements were frequently observed, mostly concentrated in biofilm structures or heterogeneously spread on the septum surface (Fig. 3). On asymptomatic septa, bacterial elements could be observed in biofilm-like structures for one sample (Fig. 3B, sample 2) but overall occurred sparsely. Generally, bacterial biomass appeared lower in asymptomatic samples. In addition to bacterial elements, the nucleic acid stains also bound to host cell nuclei (Fig. 3). Taken together, these results indicate that symptomatic TIVAPs are characterized by higher bacterial biomass with evidence for biofilm-like structures but low species richness. In contrast, although asymptomatic TIVAPs display higher species diversity, bacterial biomass is low with little or no evidence of biofilm structures.

10.1128/mSphere.00146-17.4 TABLE S3  Bacteria detected on chamber septa from Limoges Hospital. Cell content on the TIVAP septum surface was stained after freeze-thaw treatment with SYTO9 (green, live cells) and propidium iodide (red, membrane-compromised cells) from the LIVE/DEAD BacLight bacterial viability kit and imaged under ×40 magnification with a Nikon epifluorescence microscope. A minimum of 20 fields of view per sample was analyzed for elements consistent with bacterial cell shape and size. Download TABLE S3, PDF file, 0.1 MB. Copyright © 2017 Stressmann et al. 2017 Stressmann et al. This content is distributed under the terms of the Creative Commons Attribution 4.0 International license.

### Bacteria detected in culture-negative samples differ between the two tested hospital cohorts.

**FIG 4:**
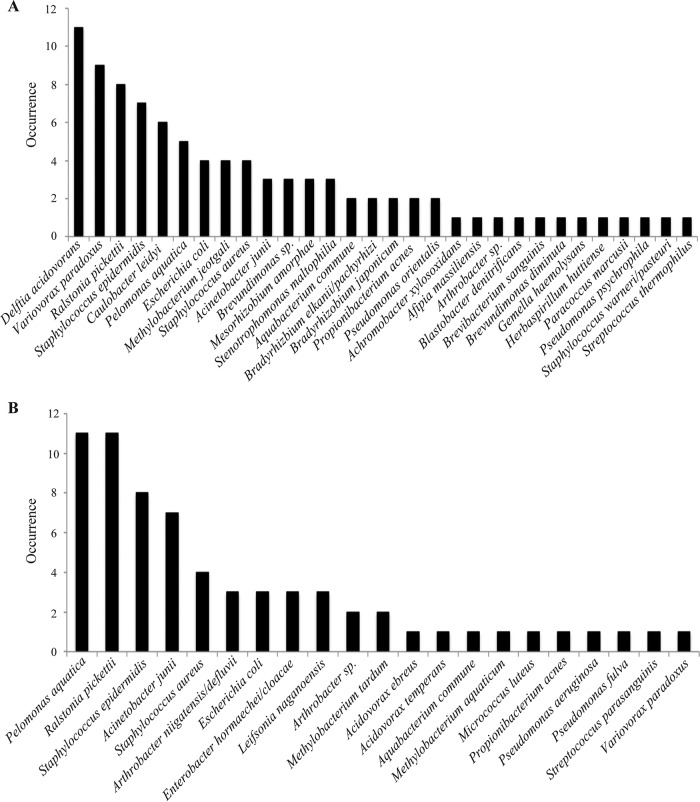
Species occurrence in all TIVAP chambers from Curie (A) and Limoges (B) Hospitals. Number of times that clones from a particular species/OTU were detected in each hospital group.

To study potential similarities among species identified in asymptomatic TIVAPs collected in the two hospitals, we compared species richness and occurrence. We observed that mean species richness in Curie Hospital asymptomatic TIVAP chambers (6.3 ± 2.5 species/sample, range of 3 to 10) was not significantly different (*P* = 0.06) from that in Limoges Dupuytren Hospital asymptomatic TIVAPs (3.1 ± 2.6 species/sample, range of 2 to 8). However, from species detected in asymptomatic TIVAPs, only 18% (*A. junii*, *Arthrobacter* sp., *E. coli*, *P. aquatica*, *P. acnes*, *R. pickettii*, and *S. epidermidis*) occurred in both hospitals, while 52% and 30%, respectively, occurred in Curie or Limoges Hospital samples only. The species occurring in both hospitals’ asymptomatic TIVAP samples were also all found in symptomatic samples. We then compared the origins of the detected species in our samples. In Limoges Hospital, the five most frequently occurring species in symptomatic chambers were associated with infection (*S. epidermidis*, *S. aureus*, *A. junii*, and *Enterobacter hormaechei*, in decreasing order of occurrence) with the exception of *Arthrobacter niigatensis*. In asymptomatic samples from Limoges, the five most frequently occurring species were of mixed origin (infection associated, *R. pickettii*, *A. junii*, and *S. epidermidis*; environmental, *P. aquatica* and *A. niigatensis*). In Curie Hospital, the five most frequently detected species in symptomatic samples were also of mixed origin (infection associated, *S. epidermidis*, *S. aureus*, and *E. coli*; environmental, *Aquabacterium commune* and *D. acidovorans*), whereas in asymptomatic samples all five most frequently encountered species were of environmental origin (*D. acidovorans*, *V. paradoxus*, *C. leidyi*, *Brevundimonas nasdae*, and *Mesorhizobium amorphae*) (Fig. 4).

These results therefore show that while species richnesses were similar between asymptomatic samples from the hospitals, species composition varied significantly, and no bacterial species assemblage specific to asymptomatic samples could be identified for both sample cohorts.

### Correlation between clinical and microbiological data.

In order to test whether any clinical parameters correlated with microbiological results (culture or 16S rRNA clone library data), a total of 38 variables (17 clinical and 21 microbiological, Table S4) were regressed with the nonparametric Pearson correlation coefficient. As the number of multiple comparisons was high and to eliminate false-positive results, Bonferroni corrections were carried out. After correction, only 6 significant correlations were found. Implant duration strongly correlated with the reason for catheter removal (i.e., the earlier that a catheter was removed, the more likely that it was due to infection, but no correlation was found with any tested microbial community parameters) (*P* < 0.0006, *r*^2^ = −0.54). Moreover, the lower the species richness (*P* = 0.0001, *r*^2^ = −0.569) and the higher the percentage of the dominant species (*P* < 0.0001, *r*^2^ = 0.721), the more likely catheters were to be removed for suspicion of infection. This was in particular the case for *S. aureus* (*P* < 0.0001, *r*^2^ = 0.639). Species richness negatively correlated with the abundance of the dominant species detected by the 16S rRNA-based approach (*P* < 0.0001, *r*^2^ = −0.823), i.e., the higher the abundance of the dominant species, the lower was the species richness. Finally, the detection of four OTUs was found to be correlated. When *R. pickettii* was identified, *P. aquatica* was likely to be found in the same sample (*P* < 0.0001, *r*^2^ = 0.763), and when a *Pseudomonas* species was identified, it was also likely to be found with an *Acinetobacter* species in the same sample (*P* < 0.0001, *r*^2^ = −0.88).

10.1128/mSphere.00146-17.5 TABLE S4  Variables used for correlation analysis between clinical and microbiological parameters. Variables were regressed with a nonparametric Pearson correlation coefficient, and Bonferroni corrections were carried out. Download TABLE S4, PDF file, 0.1 MB. Copyright © 2017 Stressmann et al. 2017 Stressmann et al. This content is distributed under the terms of the Creative Commons Attribution 4.0 International license.

These results show that symptomatic samples were more likely to have low species richness and strong dominance in the community structure.

### Culture-positive and culture-negative catheters display distinct community compositions.

In order to compare the relative diversities for symptomatic and asymptomatic samples, we calculated both Simpson and Shannon diversity indices. Both diversity index values indicated a slightly higher relative diversity for asymptomatic samples (Simpson = 0.83, Shannon = 2.32) than for symptomatic samples (Simpson = 0.77, Shannon = 1.92) (Table S5). However, there was no significant difference between the two groups (*P* = 0.63 and 0.74 for Simpson and Shannon diversity, respectively).

10.1128/mSphere.00146-17.6 TABLE S5  Diversity and evenness indices for symptomatic and asymptomatic samples from both hospitals calculated from 16S clone library data. Download TABLE S5, PDF file, 0.1 MB. Copyright © 2017 Stressmann et al. 2017 Stressmann et al. This content is distributed under the terms of the Creative Commons Attribution 4.0 International license.

**FIG 5:**
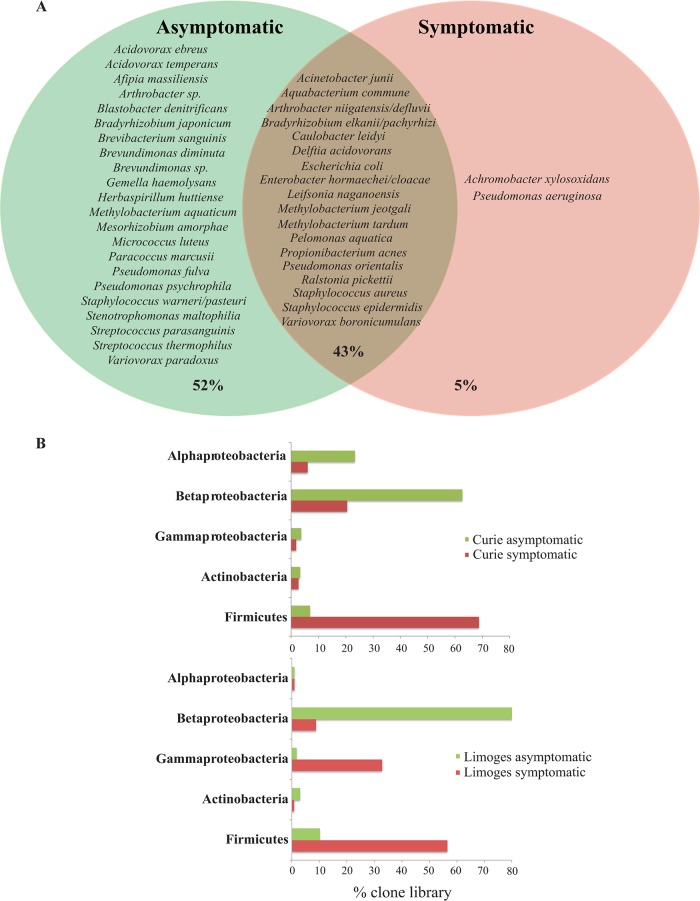
Species occurrence and phylum distribution in symptomatic and asymptomatic samples from both hospitals. (A) Species occurrence. The Venn diagram shows species (OTUs) occurring in either sample group alone or in both sample groups (brown-shaded area). Percentages indicate the number of OTUs under each condition compared to the overall total. (B**)** Phylum distribution.

In addition to relative diversity, we also compared species and phylum composition between symptomatic and asymptomatic TIVAPs to determine whether it could reflect TIVAP infection history. 16S rRNA-based analysis revealed that only 5% of the species detected (*A. xylosoxidans* and *P. aeruginosa*) occurred in symptomatic TIVAPs only, i.e., were specific to infection, while 52% of species were detected in only asymptomatic samples and 43% of species occurred in both types of TIVAPs (Fig. 5A). Comparison of phylum distributions of the detected species showed that symptomatic TIVAP samples contained markedly more species belonging to the *Firmicutes*, whereas asymptomatic samples contained many more species belonging to the *Betaproteobacteria* (Fig. 5B).

Hierarchical cluster analysis using the Bray-Curtis similarity index was carried out to determine how similar symptomatic and asymptomatic chambers were in terms of bacterial presence and abundance (Fig. S1). Strong clustering of symptomatic TIVAP samples from both hospitals could be observed, which was due to samples where either a single or a highly dominant species was detected. In contrast, asymptomatic samples formed much looser clusters and appeared overall more dissimilar to each other. The majority of Limoges asymptomatic samples clustered more tightly than Curie asymptomatic samples due to the relatively high abundance of *P. aquatica* and *R. pickettii* in these samples.

10.1128/mSphere.00146-17.1 FIG S1  Hierarchical cluster analysis of bacterial community similarity in both hospital sample groups. The Bray-Curtis index was used to calculate similarity in bacterial presence and abundance between all samples in this study. Patient numbering: CS, “symptomatic” TIVAP from Curie Hospital; CA, “asymptomatic” TIVAP from Curie Hospital; LS, “symptomatic” TIVAP from Limoges Hospital; LA, “asymptomatic” TIVAP from Limoges. Download FIG S1, PDF file, 0.2 MB. Copyright © 2017 Stressmann et al. 2017 Stressmann et al. This content is distributed under the terms of the Creative Commons Attribution 4.0 International license.

These results therefore show no significant difference in species diversity but reveal a distinct community composition pattern distinguishing symptomatic and asymptomatic TIVAP samples at the phylum level. However, such a distinctive pattern could not be distinguished on a species level.

### Difference in community structure revealed by culture-independent analysis is not an indicator of individual TIVAP clinical status.

**FIG 6:**
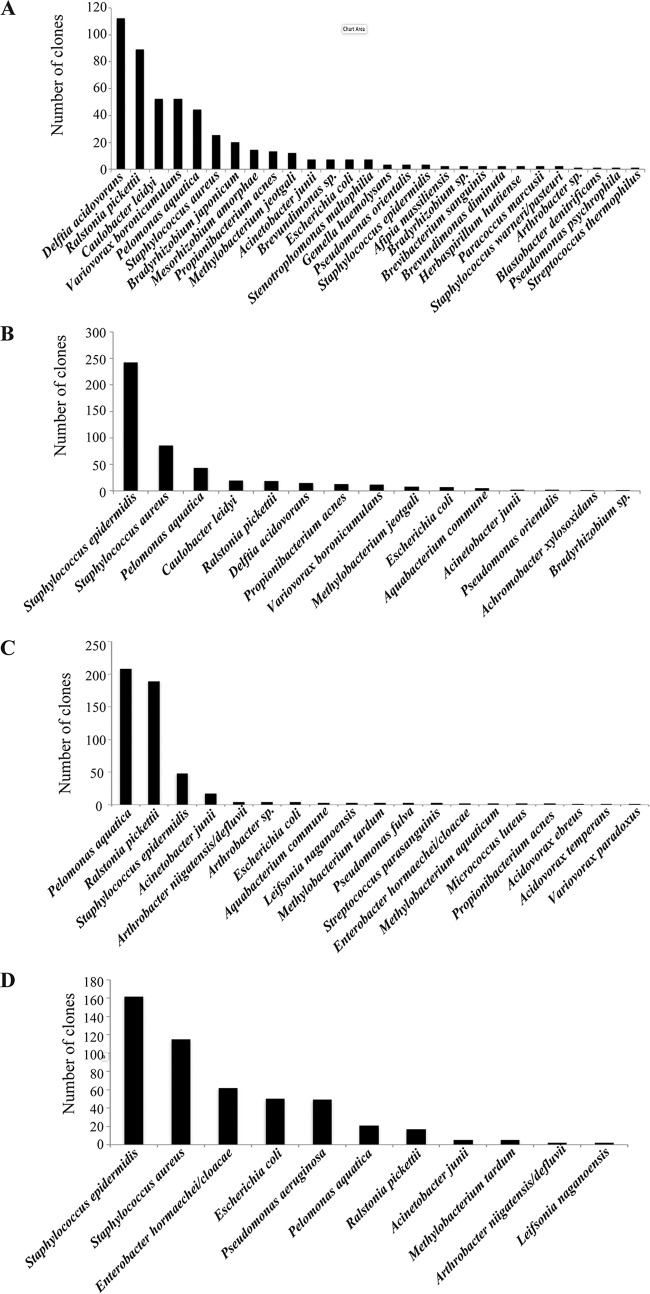
Species abundance rank order. Species/OTUs are shown ranked according to their clone library abundance in the sample group. (A) Curie asymptomatic samples; (B) Curie symptomatic samples; (C) Limoges asymptomatic samples; (D) Limoges symptomatic samples.

In order to test whether, if not community composition, community structure could differentiate asymptomatic and symptomatic TIVAPs, we plotted species rank abundance curves. The five most abundantly detected species in Limoges Hospital symptomatic TIVAPs were of clinical origin and corresponded to species also detected by culture (Fig. 6). In asymptomatic Limoges TIVAP samples, the five most abundant species included two species associated with infection (*S. epidermidis* and *A. junii*) and three of environmental origin. In Curie Hospital, the five most abundant species in symptomatic chambers included two species associated with clinical infection (the two main culture-detected species), as well as three species of environmental origin, whereas in Curie Hospital asymptomatic samples, all five most abundant species were of environmental origin. This demonstrates that the species detected as most abundant by 16S rRNA PCR and sequencing differed between hospitals and sample groups and did not allow the distinction between symptomatic and asymptomatic TIVAPs.

**FIG 7:**
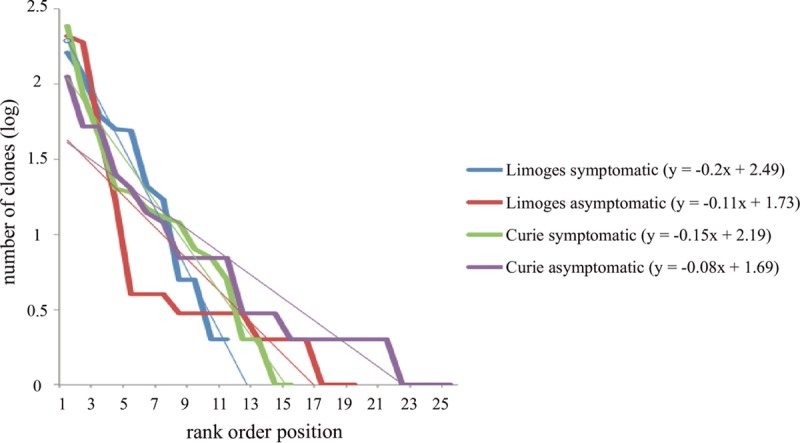
Slopes of the rank abundance curves for all sample groups. For each sample group, bacterial species were ranked according to their frequency in the clone library and the slope was calculated for this rank abundance curve.

In order to compare community structures, we calculated total species evenness (*E*) for symptomatic and asymptomatic samples and the mean slope of the rank abundance curves for each sample group (Fig. 7). Asymptomatic samples showed species evenness (*E* = 0.63) similar to that of symptomatic samples (*E* = 0.64), but the mean slope for symptomatic samples was two times steeper (Curie, *m* = 0.149; Limoges, *m* = 0.203) than for asymptomatic samples (Curie, *m* = 0.077; Limoges, *m* = 0.105). When looking at individual sample rank abundance curve slopes, there was no statistical difference (*P* > 0.05) between symptomatic and asymptomatic samples in Curie Hospital or between symptomatic samples in the two hospitals. However, the slopes of individual asymptomatic and symptomatic samples in Limoges Hospital (*P* = 0.02), as well as asymptomatic samples in the two hospitals, differed significantly (*P* = 0.03).

These results therefore indicate that symptomatic and asymptomatic sample communities were similarly even. The shape of the rank abundance curve, i.e., community structure, could distinguish between symptomatic and asymptomatic TIVAPs on a sample group but not an individual sample level.

## DISCUSSION

We studied the bacterial colonization status of TIVAPs suspected or not of infection in two French hospitals. Whereas routine microbiology analysis yielded negative culture results for almost all asymptomatic samples, culture-independent 16S rRNA gene clone library generation identified bacteria as present in all clinically symptomatic as well as asymptomatic chambers. This is consistent with the literature showing that, irrespective of development of infection, devices become colonized after insertion (12, 15). Overall, culture and molecular data corresponded well. The latter confirmed the species identified by culture as dominant OTUs in all but 5 cases. In these samples, the cultured organism was detected in very low numbers. It is likely that these organisms were outcompeted or underrepresented due to PCR bias by OTUs not selected for under the culture conditions.

We identified a total of 42 different OTUs, indicating that catheter hubs were colonized by relatively complex bacterial communities. Two previous reports using molecular techniques to investigate colonization of symptomatic and asymptomatic intravascular devices also found a high number of OTUs in asymptomatic samples (13, 16). The species richness that we identified in our study was comparable to that identified by Larsen et al. (17). However, studies using high-throughput sequencing to analyze bacteria on intravascular devices found significantly higher numbers of OTUs (e.g., reference 13 identified an average of 100 OTUs per sample on peripheral venous catheters and reference 18 identified a total of 123 OTUs on needleless connectors for central venous catheters). This is not surprising, as a much greater sequencing depth and detection of low-abundance species are to be expected with these techniques.

Contamination of intravascular devices, especially long-term indwelling devices that are frequently accessed, can occur via the skin of the patient, the hospital environment, or contaminated fluids (13, 19). Consistently, the species that we identified in symptomatic chambers were indeed skin-associated flora, as well as human gut-associated species, hospital-associated species, and species considered of environmental origin. In asymptomatic samples, skin and clinical species were also frequently detected; however, the majority of species were of environmental origin.

A number of the species identified in our study and considered of environmental origin were also detected in previous studies using molecular approaches to analyze intravascular devices. For example, *Delftia acidovorans*, the most frequently found species in asymptomatic patients from Curie Hospital, was detected by Zhang and colleagues (16). However, many of the species identified in our study have not previously been reported in catheter-related infections (e.g., *Blastobacter denitrificans*, *Herbaspirillum huttiense*, and *Variovorax paradoxus*).

In long-term indwelling devices such as TIVAPs, contamination of the chamber or hub is the most common cause of device-related infections (1, 20). We detected bacteria in TIVAP chambers of all samples, both symptomatic and asymptomatic, in both hospitals by 16S rRNA clone library generation. However, biomass appeared to be lower in asymptomatic than in symptomatic samples. This is consistent with quantitative electron microscopic studies (12) that suggest that indeed most indwelling devices become colonized (to various degrees) after insertion and that only a low percentage of implants become infected (21). A number of other studies also detected bacteria by culture-independent means in samples that were culture negative (13, 16–18). However, it remains difficult to determine whether these bacteria represent true colonization of the TIVAP chamber or are simply transients or reflect the presence of DNA from dead (e.g., due to use of antibiotics) bacteria.

Considering the sensitivity of molecular techniques to contamination, we took great care to identify and exclude potential contamination. When species found solely in reagents or materials used for molecular processing were also found in patient samples, these were excluded from analysis. However, *Pelomonas aquatica*, *Ralstonia pickettii*, and *Propionibacterium acnes* were frequently detected in patient samples in our study and were also detected as present in manipulated nonimplanted chambers and the NaCl solutions used by hospital staff to flush the chambers in order to remove the bacteria. We included these species in our analysis, as they were also detected by molecular means as dominant species in indwelling devices in references 13 and 18, respectively, and *Ralstonia pickettii* has indeed been shown to be a common contaminant of hospital solutions (22, 23). As these species were not detected in nonimplanted nonmanipulated chambers but in nonimplanted chambers that were also flushed with the routinely used NaCl solution, it is uncertain whether these species could have been introduced as contaminants during sample processing or were already present in the flush solutions or whether the detection of such important levels of these species in our study represents true colonization. Furthermore, we could not analyze the solutions that were routinely introduced into the TIVAP chambers while still implanted. Therefore, we cannot draw any conclusions on potential introduction of these contaminants during routine procedures. However, it is generally possible that species potentially present in these routine working solutions could contribute significantly to the final community composition and structure as detectable by PCR-based methods, especially in the absence of a dominant pathogen such as is the case for most of the asymptomatic chambers. Our analysis is thus calling for increased attention to this problem.

We detected significantly more species by 16S rRNA clone library generation in asymptomatic chambers than in symptomatic samples. However, the Simpson and Shannon relative diversity indices did not show this difference to be significant. Three previous molecular studies of bacterial presence on “colonized” and “uncolonized” arterial and central venous catheters (13, 16) also found no difference in relative diversity between the two groups; however, they did not detect a difference in OTU richness between the two sample types (13, 16). This could be due to truly a higher number of species being present in asymptomatic chambers in our study or could be a technical artifact. The lower bacterial biomass and absence of a highly dominant OTU in asymptomatic chambers could have led to a higher detection of different (rare) OTUs in these samples due to PCR bias (24). Due to small template amounts, we were unable to carry out quantitative confirmation such as real-time quantitative PCR or fluorescent *in situ* hybridization (FISH) to investigate this; however, in future studies quantitative techniques should be integrated into the molecular analysis.

Our study found little overlap in bacterial presence on symptomatic and asymptomatic TIVAPs between the two French hospitals. We studied whether there was a potential common pool of bacteria colonizing asymptomatic TIVAPs that did not trigger infection. We found that only 18% of species occurred in asymptomatic chambers from both hospitals, all of which also occurred in symptomatic chambers. Further, the five most frequently occurring species also differed between the two asymptomatic sample cohorts. It has previously been shown that bacterial presence in different patient populations can vary dramatically between hospitals or even hospital sites and is observed in many complex polymicrobial colonization scenarios (e.g., gut [25], prosthetic joint infection [26], and wound infections [27]). However, the hospital-specific bacterial community patterns prevented us from drawing generalizable conclusions regarding the presence of bacteria in symptomatic and asymptomatic chambers.

The diagnosis of vascular device-associated infections is notoriously difficult (28). A better understanding of the pathogenic potential of any given vascular device would therefore be crucial to improving clinical practice. Microscopy and a number of more recent molecular studies have built the case that vascular devices with and without symptoms are both colonized by a variety of microorganisms (12, 13, 16, 18). However, although colonization is a prerequisite for infection, it does not in itself appear to be sufficient to predict infection, as only a low percentage of catheters become infected (12). What leads to catheter infection therefore remains unknown, and different theories have been proposed. For example, bacterial diversity in itself may promote increased pathogenicity or persistence in chronic infections (29, 30). Brogden et al. (31) suggested that in a polymicrobial infection, microorganisms may act synergistically and present different clinical outcomes than those in infection by a single organism. In our study, we were particularly interested in whether we could identify a bacterial community parameter or signature indicative of the clinical status or fate of an implant that could potentially serve as a marker for infection.

As a predictive marker should be easily detectable and interpretable, we first investigated whether the most abundantly detected species allowed distinction between symptomatic and asymptomatic samples. In neither hospital did the most abundant species allow clear distinction between either sample group; moreover, the species detected were partly hospital specific and did not allow a cross-site sample distinction either. When expanding the analysis to all species detected, we identified only 5% of species that were exclusively present in symptomatic TIVAP chambers, while other organisms, commonly associated with infection, interestingly occurred in both symptomatic and asymptomatic chambers. This is consistent with the previous findings that the simple presence of a species known to cause infection does not necessarily predict infection. We found that 52% of identified species, of mostly environmental origin, occurred in asymptomatic chambers alone. We were interested whether this could be used to predict the infection potential, e.g., whether an implant with any of these species would be more or less likely to present with clinical infection symptoms. We identified strong sample clustering, based on the presence of highly abundant hospital-associated species in symptomatic samples, and also found that the presence of highly abundant *P. aquatica* or *R. pickettii* led to some clustering in asymptomatic samples in Limoges which could be interesting for future studies. However, weak cross-site sample clustering and weak similarity between hospitals prevented the identification of a specific species assemblage consistently indicative of clinical status in our study. Comparison of species presences at the phylum level identified an increased presence of species belonging to the *Firmicutes* in symptomatic samples and a higher percentage of *Betaproteobacteria* in asymptomatic samples. This is interesting, as an increase in *Firmicutes* members has been described for other infection scenarios such as in the gut (32) and should be investigated in future studies.

Finally, we investigated community structure as it was shown to be associated with clinical status in different diseases (33–36), where decreased evenness can be indicative of an imbalance, e.g., an infection, in the community. We found that, when analyzed as a group, asymptomatic samples showed similar total evenness as symptomatic samples but that in both hospitals the asymptomatic samples showed a rank abundance curve slope that was only half as steep as that for symptomatic samples. However, when rank abundance slopes were analyzed on an individual sample basis, as would be the case for a diagnostic prediction marker, they were not able to distinguish consistently between symptomatic and asymptomatic samples. This could, however, be interesting to investigate in future studies with a bigger sample group drawn from a number of different hospital sites.

Overall, our study of the bacterial colonization status of TIVAPs suspected or not of infection in two French hospitals confirms previous findings that both symptomatic and asymptomatic TIVAP chamber implants appear to be colonized by bacteria. Although we did not identify a particular species assemblage consistently predictive of infectious status of an implant, we identified a difference between symptomatic and asymptomatic samples in species richness, phylum composition, and community structure on a sample group level, suggesting that the microbial ecology of intravascular devices could be predictive of TIVAP infection susceptibility. Hence, this improves our understanding of the nature and evolution of intravascular device-associated infections. Further investigations combining culture techniques, quantitative sequencing analyses, and microscopic techniques could provide insight into the colonization dynamics of central venous catheters. Ultimately, this may lead to the definition of a microbial ecology signature that can predict infection susceptibility of individual medical implants.

## MATERIALS AND METHODS

### Patient background.

Totally implantable venous access port (TIVAP) systems (Celsite [B. Braun, Melsungen, Germany] and Heliosite [Vygon, Paris, France]) were collected from the Anesthesia-Reanimation-Pain unit at the Institut Curie Hospital (Paris) between October 2008 and September 2009 and from the surgery department at the Limoges Dupuytren Hospital between August 2009 and July 2012. The medical personnel performing the TIVAP removal gave information and obtained the patient’s oral consent for sample collection.

All patients had TIVAP implants for cancer chemotherapy (see Table S1 in the supplemental material). “Asymptomatic” patients (*n* = 20, 10 from each hospital) had a minimum implant duration of 6 months and were selected on the basis of absence of signs of infection from implantation to removal and absence or a minimum administration of antibiotic treatments during the last 6 months of implant indwelling. The criterion for TIVAP removal for “asymptomatic” patients (*n* = 20, 10 from each hospital) was the end of treatment in the absence of suspected catheter-associated infection. We did not perform blood cultures for “asymptomatic” patients because such analysis is not included in the standard-of-care for such situations. “Symptomatic” patients were enrolled on the basis of suspicion of TIVAP implant infection, notably local and/or systemic clinical signs of infection and positive blood culture. TIVAP removal for “symptomatic” patients was based on the clinical decision of the treating physician in response to the suspected infection, with or without administration of antibiotics.

The patient group at Curie Hospital consisted of 7 males and 13 females, with a mean age of 52.3 ± 18.9 years and a mean TIVAP implant period of 101.7 days for infected and 449.7 days for noninfected TIVAPs. The patient group at Limoges Dupuytren Hospital consisted of 8 males and 12 females, with a mean age of 62.5 ± 10.7 years and a mean TIVAP implant period of 116.3 days for infected and 356.6 days for noninfected TIVAPs (Table S1). No significant associations were observed between TIVAP colonization and antibiotic treatment in the 4 weeks prior to TIVAP removal.

### Sample collection.

Inpatient TIVAP implantation, handling, and TIVAP removal were carried out under routine aseptic hospital protocols by experienced surgical staff. After removal, TIVAPs were immediately transferred to the microbiology laboratories of the respective hospitals and handled under laminar flow hoods with sterile reagents and instruments. Special arrangements were made with the hospitals to also process the noninfected TIVAPs, which are not routinely analyzed. In accordance with one of the methods for bacterial isolation from TIVAPs recommended by the European Society for Clinical Microbiology and Infectious Disease, the TIVAP chamber/hub was separated from the TIVAP catheter, disinfected on the outside with a pad soaked in povidone-iodine, and flushed with 1 ml of sterile saline solution (37, 38). This flush solution was then used for routine diagnostic culture, and 700 μl of the flush was immediately stored at −80°C for transfer to the Institut Pasteur and nucleic acid extraction. With each sample batch, an aliquot of the NaCl solution used to flush the chambers after explantation was also collected as reagent control. Control TIVAPs (nonimplanted nonmanipulated chambers and nonimplanted chambers that were manipulated like real samples, including the flush with saline solution) were collected to determine potential sources of bacterial contamination.

### Routine diagnostic culture.

The chamber flush was diluted and spotted on Columbia blood agar and chocolate agar for quantitative monitoring and also inoculated into liquid brain heart infusion medium and incubated for 24 to 48 h at 37°C under aerobic conditions. Identification and quantification of growing bacteria were carried out according to hospital standard operating procedures by hospital microbiologists and confirmed with Vitek mass spectrometry (MS) (matrix-assisted laser desorption ionization–time of flight [MALDI-TOF]).

### DNA extraction from clinical samples.

Prior to sample processing, 4 DNA extraction methods (QIAamp DNA minikit, Promega Wizard genomic extraction kit, Mo Bio PowerLyzer Ultraclean DNA isolation kit, and guanidium thiocyanate-EDTA-Sarkosyl extraction) were evaluated. The method with the best results for equal lysis of Gram-negative and Gram-positive bacteria, highest DNA yields from low starting material, and lowest intrinsic bacterial contamination was determined to be the Mo Bio PowerLyzer Ultraclean kit. Bacterial DNA was extracted from the TIVAP chamber flush liquid according to the manufacturer’s instructions. Control (DNA-free water as the template) extraction samples were run with each extraction in order to identify any potential DNA traces introduced during the extraction procedure. Extracted genomic DNA was verified by Tris-acetate-EDTA-agarose gel electrophoresis (1%), stained with GelRed, and quantified by applying 2.5 μl directly to a NanoDrop ND-1000 spectrophotometer.

### 16S rRNA gene analysis—PCR and clone library generation.

The universal primers for the domain *Bacteria*, 8f (5′-AGA GTT TGA TCC TGG CTC AG-3′) and 1492r (5′-GGT TAC CTT GTT ACG ACT T-3′), were used to amplify the bacterial 16S rRNA gene. Each primer was used at a final concentration of 0.2 μM, and 25 to 50 ng of DNA was added as the template to 50-μl reaction mixtures. PCR cycling conditions were as follows: initial denaturation at 94°C for 2 min, followed by 32 cycles of denaturation at 94°C for 1 min, annealing at 56°C for 1 min, and extension at 72°C for 2 min, with a final extension step at 72°C for 10 min. 16S rRNA gene PCR products were verified on 1% agarose gels, purified with the QIAquick PCR purification kit, and cloned with the pGEM-T Easy vector system (Promega) according to the manufacturer’s instructions. The presence of the cloned insert was confirmed by colony PCR with vector primers gemsp6 (5′-GCT GCG ACT TCA CTA GTG AT-3′) and gemt7 (5′-GTG GCA GCG GGA ATT CGA T-3′). Clone PCR products with an insert of the correct size were purified as described above and sent for sequencing (Eurofins, Ebersberg, Germany). All reagents used for TIVAP flushing, DNA extraction, PCR, and clone library generation were subjected to 16S rRNA sequencing (Fig. 1A, green asterisks indicate all protocol steps at which reagents were tested). Materials (e.g., tubes) were flushed with DNA-free water and also sequenced (Fig. 1A, blue asterisks). Blanks using DNA-free water as the template were run for all procedures to control for handling and equipment contamination and were also subjected to 16S rRNA sequencing (Fig. 1A, red asterisks).

Clone library coverage was calculated by the formula [1 − (*n*_1_/*N*_2_)] × 100, where *n*_1_ is the number of singletons detected in the clone library and *N*_2_ is the total number of clones generated for this sample. Clone libraries were generated to a minimum coverage of 95%, and 960 and 988 clones were generated for clinically symptomatic and clinically asymptomatic TIVAPs, respectively, from both hospitals (excluding low-quality sequences and contaminants).

### Sequence analysis.

16S rRNA sequences were manually proofread. Sequences with ambiguous bases or low quality were removed from the analysis. Primer sequences were trimmed off, and sequences were compared to GenBank (NCBI) with BLAST and to the Ribosomal Database Project with SeqMatch. A 98% similarity cutoff was used for operational taxonomic unit (OTU) determination, and a 95% cutoff was used for genus determination.

### Sample staining and microscopy.

Eight TIVAP chamber septa were collected separately from Limoges Hospital; four septa originated from clinically symptomatic patients, and four were from clinically asymptomatic patients. Septa were removed from their respective TIVAP chambers under sterile conditions. The septum side residing in the chamber was stained with approximately 10 μM SYTO9 and approximately 50 μM propidium iodide nucleic acid stains (LIVE/DEAD BacLight bacterial viability kits; Molecular Probes, Invitrogen) and incubated for 15 to 60 min in the dark.

Septa were imaged using a Nikon DN100 digital network camera mounted on a Nikon Eclipse E400 epifluorescence microscope equipped with a 40× 0.75-numerical-aperture (NA) Plan Fluor objective. To excite and visualize labeled samples, a 100-W super-high-pressure mercury lamp was utilized and filtered through a green fluorescent protein (GFP) long-pass filter cube (482-/35-nm excitation filter, 505-nm dichroic mirror, 510-nm barrier filter) (Nikon France SAS). An exposure time of 17 ms was used, and a minimum of 20 fields of view was acquired for each sample. Images were manually assayed for the presence of bacteria; bacterial cells and human cells were distinguished by morphology and size.

### Bacterial community and statistical analysis.

We used bacterial occurrence as the determination of the presence or absence of an OTU independent of its abundance in the clone library and bacterial dominance as the abundance of an OTU either within a sample or within a sample group. Rank abundance curves were constructed by ranking the abundance of OTUs (number of clones) in a sample or sample set from most to least abundant. Linear regression was used to calculate the equation for the rank abundance curve (*y* = *mx* + *p*) where *m* is the slope of the rank abundance curve.

The Simpson diversity index was calculated as the sum of the relative species abundance (*P*) squared (*D* = Σ*P*^2^), and the Gini-Simpson index (1 − *D*) was used in this paper in order to use positive correlation values. The Shannon diversity index was calculated with the formula *H*_*S*_ = −Σ{*P*[ln(*P*)]}, where *P* is the relative species abundance. Total evenness was calculated for the Shannon index as *E* = *H*_*S*_/*H*_max_.

Community similarity between samples was assessed by hierarchical cluster analysis using the Bray-Curtis similarity index [2*jN*/(*N*_*a*_ + *N*_*b*_)], where *N*_*a*_ is the total number of OTUs in sample 1, *N*_*b*_ is the total number of OTUs in sample 2, and 2*jN* is the sum of the lower of the two abundances for species found in both sites.

Two-tailed *t* tests for the comparison of two data sets were carried out in SPSS software, version 20 (Chicago, IL), with *P* being considered significant when it was <0.05. Correlation analysis was also carried out in SPSS using Pearson’s correlation coefficient *R* to determine linear relationships between clinical and microbiological variables and their significance *P*. Bonferroni corrections were applied to eliminate false-positive results due to the high number of multiple comparisons.

### Accession number(s).

Sequences have been deposited in GenBank under accession numbers MF377637 to MF378601 and MF399825 to MF400811.
