# Supplementary material for: Comparative Analysis of Bacterial Community Composition and Structure in Clinically Symptomatic and Asymptomatic Central Venous Catheters
Source: mSphere. 2017 Sep 27;2(5):e00146-17. doi: 10.1128/mSphere.00146-17 (PMC5615130; doi:10.1128/mSphere.00146-17)
Supplement: TABLE S1 [file sph005172363st2.pdf]

Sample ID	Sex	Age	Reason for catheter implant	Implant duration (days)	Catheter infection in the last 6 months	Antibiotics <4 weeks prior to catheter removal	Parenteral nutrition	Reason for catheter removal
CS-1	F	50	Breast cancer	135	No	Tavanic	No	Infection suspicion
CS-2	M	57	Bronchial cancer	88	Yes	N/A	No	Infection suspicion
CS-3	M	34	Osteosarcoma	unknown	No	Vancomycin	No	Infection suspicion
CS-4	F	58	Breast cancer	unknown	No	Amoxicillin/Ciprofloxacin	No	Infection suspicion
CS-5	M	50	ORL carcinoma	146	Yes	Vancomycin/Gentamicin	No	Infection suspicion
CS-6	F	13	Ewing tumor	unknown	No	Caspofungin	No	Fungal infection
CS-7	F	18	Pineal gland tumor	unknown	No	Targocid	No	Infection suspicion
CS-8	F	74	Breast cancer	22	No	Amoxicillin	No	Infection suspicion
CS-9	M	63	Bronchial carcinoma	27	No	Amoxicillin/Ofloxacin	No	Infection suspicion
CS-10	M	14	Naso-pharyngeal carcinoma	192	No	Vancomycin/voriconazole	No	Infection suspicion
CA-1	F	60	Breast cancer	750	No	N/A	No	End of treatment
CA-2	F	65	Breast cancer	269	No	N/A	No	End of treatment
CA-3	M	66	Pulmonary adenocarcinoma	184	No	N/A	No	End of treatment
CA-4	F	68	Breast cancer	186	No	N/A	No	End of treatment
CA-5	F	69	Breast cancer	535	No	N/A	No	End of treatment
CA-6	F	68	Lymphoma	276	No	N/A	No	End of treatment
CA-7	F	38	Gastro-adenocarcinoma	1324	No	N/A	No	End of treatment
CA-8	F	63	Breast cancer	481	No	N/A	No	End of treatment
CA-9	F	54	Breast cancer	261	No	N/A	No	End of treatment
CA-10	M	64	Pleural thymome tumor	231	No	N/A	No	End of treatment
LS-1	F	76	Stomach adenocarcinoma	125	No	Vancomycin	Yes	Infection suspicion
LS-2	F	56	Uterus neoplasia	243	No	Pristinamycin	No	Infection suspicion
LS-3	M	62	Primary epidermoid carcinoma with advanced metastasis	37	No	N/A	No	Infection suspicion
LS-4	M	51	Pulmonary adenocarcinoma	105	No	Vancomycin/Amoxicillin/clavulanic acid	No	Infection suspicion
LS-5	M	63	Pulmonary adenocarcinoma with cerebral metastasis	88	No	Ceftriaxon/Ciprofloxacin<br>Pristinamycin/Ciprofloxacin	No	Infection suspicion
LS-6	F	42	Breast neoplasia with bone metastasis	63	No	Oxacilline	No	Infection suspicion
LS-7	M	46	Epidermoid carcinoma	34	No	N/A	No	Infection suspicion
LS-8	M	71	Papillary urothelial carcinoma	7	No	Vancomycin on removal day	No	Infection suspicion
LS-9	M	73	Oropharynx neoplasia	71	No	N/A	No	Infection suspicion
LS-10	M	68	Chronic lymphoid leukemia B	390	No	*Prophylaxis*:Sulfamethoxazole/Trimethoprim/ Phenoxymethylpenicillin.<br>*Before removal:* Ceftriaxon	No	Infection suspicion
LA-1	F	54	Breast neoplasia	603	No	N/A	No	End of treatment
LA-2	F	67	Breast neoplasia	225	No	N/A	No	End of treatment
LA-3	F	43	Hodgkin lymphoma	298	No	N/A	No	End of treatment
LA-4	F	69	Breast neoplasia	217	No	N/A	No	End of treatment
LA-5	F	73	Breast neoplasia	344	No	N/A	No	End of treatment
LA-6	M	75	Pulmonary adenocarcinoma	184	No	N/A	No	End of treatment
LA-7	F	64	Breast neoplasia	357	No	N/A	No	End of treatment
LA-8	F	61	Breast neoplasia	191	No	N/A	No	End of treatment
LA-9	F	74	Bilary duct adenocarcinoma	927	No	N/A	No	End of treatment
LA-10	F	61	Breast neoplasia	220	No	N/A	No	End of treatment

*: Patient numbering – CS = “symptomatic” catheters (retrieved for suspicion of infection) from Curie Hospital, CA = “asymptomatic” catheters from Curie Hospital, LS = “symptomatic” catheters (retrieved for suspicion of infection) from Limoges, LA = “asymptomatic” catheters from Limoges
