# Supplementary material for: Comparative Analysis of Bacterial Community Composition and Structure in Clinically Symptomatic and Asymptomatic Central Venous Catheters
Source: mSphere. 2017 Sep 27;2(5):e00146-17. doi: 10.1128/mSphere.00146-17 (PMC5615130; doi:10.1128/mSphere.00146-17)
Supplement: TABLE S2 [file sph005172363st3.pdf]

A. Species detected in PCR or extraction reagents, materials and/or introduced during sample handling in the molecular laboratory: excluded from all analyses

Burkholderia fungorum

Chryseobacterium indoltheticum

Mesorhizobium metallidurans/gobiense/tarimense/tianshanense

Pseudomonas pseudoalcaligenes/alcaliphila

Sphingobium xenophagum

Sphingomonas echinoides

Tumebacillus permanentifrigoris

B. Species detected in non-implanted and non-manipulated TIVAP chambers

Afipia broomeae

Sphingomonas echinoides

C. Species detected in hospital flush solution and/or introduced during sample handling in Curie Hospital (manipulation of non-implanted TIVAP)

Alishewanella aestuarii

Flavobacterium frigidimaris

Propiniobacterium acnes

Pseudomonas fragi

Pseudomonas nitroreducens/denitrificans

Ralstonia pickettii

Sphingobium amiense

Streptococcus mitis

D. Species detected in hospital flush solution and/or introduced during sample handling in Limoges Hospital (manipulation of non-implanted TIVAP)

Acinetobacter parvum

Arthrobacter sp.

Citrus/Azadirachta chloroplast sp.

Pelomonas aquatica/puraquae

Propiniobacterium acnes

Propiniobacterium granulosum

Ralstonia pickettii

Soil bacterium TWE165
