# Supplementary material for: Comparative Analysis of Bacterial Community Composition and Structure in Clinically Symptomatic and Asymptomatic Central Venous Catheters
Source: mSphere. 2017 Sep 27;2(5):e00146-17. doi: 10.1128/mSphere.00146-17 (PMC5615130; doi:10.1128/mSphere.00146-17)
Supplement: TABLE S3 [file sph005172363st4.pdf]

**Chamber**	**Fields of view containing elements consistent with bacteria**	**Fields of view without elements consistent with bacteria**	**Total Fields of view analysed**
**Symptomatic 1 (A1)**	26	0	26
**Symptomatic 2 (A2)**	13	12	25
**Symptomatic 3 (A3)**	16	9	25
**Symptomatic 4 (A4)**	26	0	26
**Asymptomatic 1 (B1)**	5	19	24
**Asymptomatic 2 (B2)**	5	23	28
**Asymptomatic 3 (B3)**	13	13	26
**Asymptomatic 4 (B4)**	0	21	21
