# Supplementary material for: Comparative Analysis of Bacterial Community Composition and Structure in Clinically Symptomatic and Asymptomatic Central Venous Catheters
Source: mSphere. 2017 Sep 27;2(5):e00146-17. doi: 10.1128/mSphere.00146-17 (PMC5615130; doi:10.1128/mSphere.00146-17)
Supplement: TABLE S4 [file sph005172363st5.pdf]

Clinical variables	Variable type	Microbiological variables	Variable type
patient sex	Nominal	cultured organisms	Nominal
patient age	Scale	culture of S. aureus	Nominal
reason for catheter implantation	Nominal	culture of *S.epidermidis*	Nominal
implant duration (days)	Scale	culture of *E.coli*	Nominal
infection of catheter or chamber in the last 6 months	Nominal	culture of *P. aeruginosa*	Nominal
antibiotics administered <4 weeks prior to implant removal	Nominal	culture of *E. cloacae*	Nominal
levofloxacin/ciprofloxacin administration	Nominal	culture of *Candid*a spp.	Nominal
vancomycin/teicoplanin administration	Nominal	species richness (clone library)	Scale
pristinamycin administration	Nominal	rank abundance curve slope (clone library)	Scale
amoxicillin/phenoxymethylpenicillin administration	Nominal	dominant species	Nominal
oxacillin administration	Nominal	abundance of the dominant species in each clone library	Scale
ceftriaxon administration	Nominal	abundance of *Staphylococcus* spp in clone libraries	Scale
trimethroprim/sulfamethoxazole administration	Nominal	abundance of *Pseudomonas* spp. in clone libraries	Scale
caspofungin/voriconazole administration	Nominal	abundance of *E.coli* in clone libraries	Scale
clavulanic acid administration	Nominal	abundance of *Delftia* spp. In clone libraries	Scale
parenteral nutrition	Nominal	abundanceof *Caulobacter leidyia* in clone libraries	Scale
reason for implant removal	Nominal	abundance of *Acinetobacter* spp. In clone libraries	Scale
		abundance of *Methylolobacterium* spp. In clone libraries	Scale
		abundance of *Mezorhizobium* spp. In clone libraries	Scale
		abundance of *Enterobacter* spp. In clone libraries	Scale
		abundance of *Arthrobacter* spp. In clone libraries	Scale
