# Supplementary material for: Comparative Analysis of Bacterial Community Composition and Structure in Clinically Symptomatic and Asymptomatic Central Venous Catheters
Source: mSphere. 2017 Sep 27;2(5):e00146-17. doi: 10.1128/mSphere.00146-17 (PMC5615130; doi:10.1128/mSphere.00146-17)
Supplement: TABLE S5 [file sph005172363st6.pdf]

Diversity index			
**Sample group**	**Shannon**	**Gini-Simpson**	**Evenness index**
Symptomatic	1.92	0.77	0.64
Asymptomatic	2.32	0.83	0.63
