# Supplementary figures and images for: Comparative Analysis of Bacterial Community Composition and Structure in Clinically Symptomatic and Asymptomatic Central Venous Catheters
Source: mSphere. 2017 Sep 27;2(5):e00146-17. doi: 10.1128/mSphere.00146-17 (PMC5615130; doi:10.1128/mSphere.00146-17)

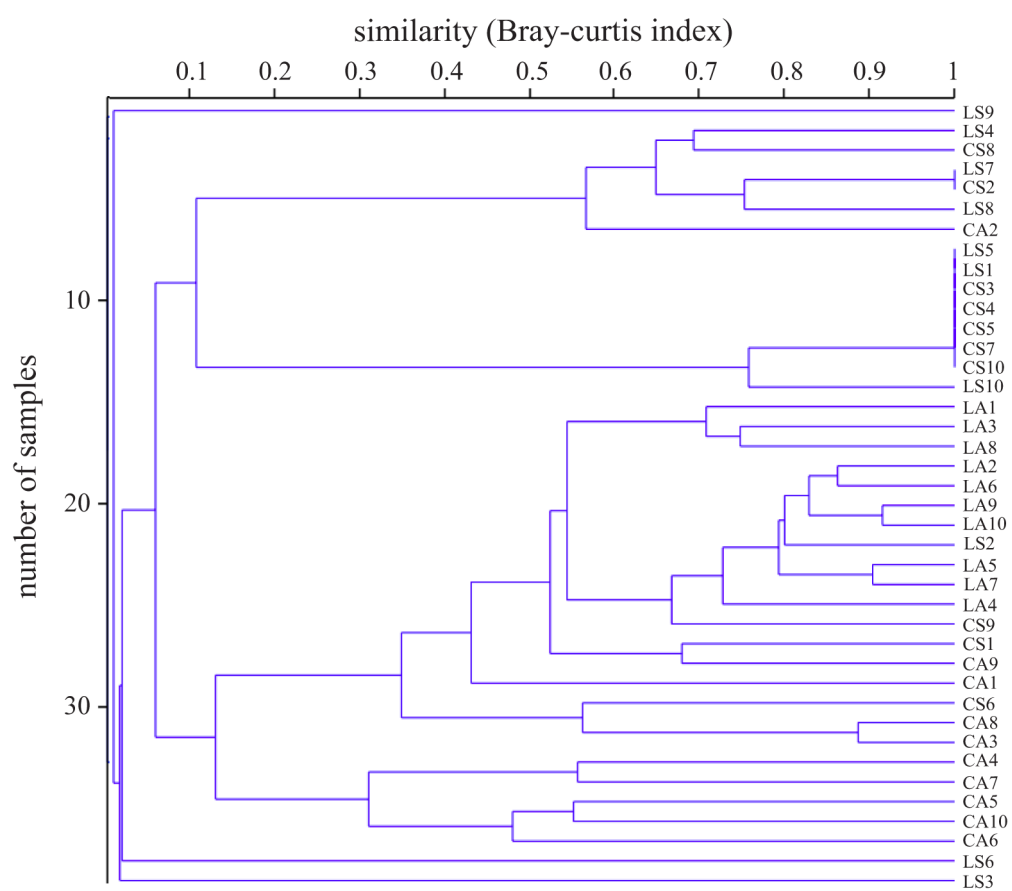

Supplement: FIG S1 [file sph005172363sf1.pdf]
